# Molecular Study of the Fukutin-Related Protein (*FKRP*) Gene in Patients from Southern Italy with Duchenne/Becker-like Phenotype

**DOI:** 10.3390/ijms251910356

**Published:** 2024-09-26

**Authors:** Antonio Qualtieri, Selene De Benedittis, Annamaria Cerantonio, Luigi Citrigno, Gemma Di Palma, Olivier Gallo, Francesca Cavalcanti, Patrizia Spadafora

**Affiliations:** Institute for Biomedical Research and Innovation, National Research Council, 87050 Mangone, Italy; antonio.qualtieri@irib.cnr.it (A.Q.); selene.debenedittis@irib.cnr.it (S.D.B.); annamaria.cerantonio@irib.cnr.it (A.C.); luigi.citrigno@irib.cnr.it (L.C.); gemma.dipalma@irib.cnr.it (G.D.P.); olivier.gallo@irib.cnr.it (O.G.); francesca.cavalcanti@irib.cnr.it (F.C.)

**Keywords:** LGMDR9, LGMD2I, limb girdle muscular dystrophy type R9, FKRP, molecular dynamic

## Abstract

Pathogenic variants localized in the gene coding for the Fukutin-Related Protein (FKRP) are responsible for Limb-Girdle Muscular Dystrophy type 9 (LGMDR9), Congenital Muscular Dystrophies type 1C (MDC1C), Walker–Warburg Syndrome (WWS), and Muscle–Eye–Brain diseases (MEBs). LGMDR9 is the fourth most common hereditary Limb Girdle Muscular Dystrophy in Italy. LGMDR9 patients with severe disease show an overlapping Duchenne/Becker phenotype and may have secondary dystrophin reduction on muscle biopsy. We conducted a molecular analysis of the *FKRP* gene by direct sequencing in 153 patients from Southern Italy (Calabria) with Duchenne/Becker-like phenotypes without confirmed genetic diagnosis. Mutational screening of the patients (112 men and 41 women, aged between 5 and 84 years), revealed pathogenic variants in 16 subjects. The most frequent variants identified were c.427C > A, p.R143S, and c.826C > A, p.L276I (NM_024301.5). The results obtained show that the Duchenne/Becker-like phenotype is frequently determined by mutations in the *FKRP* gene in our cohort and highlight the importance of considering LGMDR9 in the differential diagnosis of dystrophinopathies in Calabria. Finally, this study, which, to our knowledge, is the first conducted on Calabrian subjects, will contribute to the rapid identification and management of LGMDR9 patients.

## 1. Introduction

The *FKRP* gene, located on chromosome 19q13.3 (MIM606596), codes for the Fukutin-Related Protein (FKRP). The gene is 1.5 kb long, composed of four exons, of which only the last one represents the coding region [1].

FKRP is a type II transmembrane protein 495 amino acids long, residing in the Golgi apparatus and consisting of an N-terminal cytoplasmic domain (1–6 aa), a transmembrane hydrophobic domain (7–29 aa), a stem region (37–279 aa), and a large globular catalytic domain facing the lumen of the Golgi apparatus with a DxD motif [2,3,4,5]. The presence of this last motif, a highly conserved structure in many families of glycosyltransferases residing in the Golgi apparatus, has allowed us to hypothesize that this protein is essential for the post-translational modifications of dystroglycans [6].

This hypothesis found confirmation in Western blotting studies from muscle biopsies showing a reduction in glycosylation of alpha-dystroglycan (α-DG) in patients with mutations on the *FKRP* gene [7].

Glycosylation, which is a highly conserved post-translational modification process from fungi to humans, involves more than twenty proteins (including FKRP) in sequential action as Protein O-mannosyltransferase1 (POMT1), Protein O-mannosyltransferase2 (POMT2), Protein O-Linked Mannose N-Acetylglucosaminyltransferase1 (POMGNT1), Protein O-Linked MannoseN-Acetyl-glucosaminyltransferase2 (POMGNT2), Mannosyl (α1,6)-Glycoproteinβ1,6-NacetylGlucosaminyltransferase (MGAT5B), β-1,3 N-Acetylgalactosaminyltransferase2 (B3GALNT2), Protein O-Mannose Kinase (POMK), Fukutin protein (FKTN), Fukutin-Related Protein (FKRP), Ribitol Xylosyltransferase 1 (RXYLT1), Beta-1,4-Glucuronyltransferase1 (B4GAT1), Xylosyl- and Glucuronyltransferase LARGE1 (LARGE1), Xylosyl- and Glucuronyltransferase LARGE2 (LARGE2), Carbohydrate Sulfotransferase10 (HNK-1ST), Dolichol-Phosphate Mannosyltransferase subunit3 (DPM1-3) [8].

In the sarcolemma, the glycosylated chain of α-DG binds to the alpha-2 chain of laminin of the extracellular matrix (ECM), acting as a link between it and the intracellular cytoskeleton, protecting the sarcolemma from mechanical stress during contraction and maintaining its integrity [9].

In fact, α-dystroglycan, located outside of the plasma membrane, connects to dystrophin through β-dystroglycan (βDG). Both are part of the Dystrophin-Glycoprotein Complex (DGC), which also includes the Sarcoglycan Complex (SGC), the Dystrobrevin and Syntrophin protein families [10].

The link between the DGC and the ECM, propagates a whole series of biomechanical forces that will be converted into biochemical and/or epigenetic changes [11].

Variations in genes that code for one of the proteins forming part of this link determine a pathological alteration of the homeostasis of musculoskeletal cells and consequent onset of muscular dystrophies often associated with Dilated Cardiomyopathy (DCM). In particular, the sarcolemma of cardiomyocytes becomes particularly fragile and susceptible to mechanical stimuli with consequent dilation and progressive DCM of the heart [12].

Furthermore, many forms of muscular dystrophies are associated with brain abnormalities, ranging from mild cognitive impairment to defective neuronal migration, since the glycosylation of α-DG is essential for the normal development and function of brain tissue [13].

Pathogenic variants identified in the *FKRP* gene have been associated with a broad clinical spectrum of autosomal recessive muscular dystrophies, such as Congenital Muscular Dystrophy type 1C (MDC1C, OMIM606612), Walker–Warburg Syndrome (WWS, OMIM236670), Muscle–Eye–Brain Disease (MEB, OMIM253280) and Limb Girdle Muscular Dystrophy R9 (LGMDR9) (previously known as LGMD2I, MIM607155) [14,15,16,17,18,19].

LGMDR9 is one of the most frequent forms of LGMD in Europe, with a north-to-south genetic gradient. In Denmark, LGMDR9 comprises approximately 38% of all adult patients with LGMD. This value is reduced in Germany, Holland, and Italy to just over 4% in the Czech Republic [20].

In Italy, Limb Girdle Dystrophy R9 is the fourth most frequent form of autosomal recessive LGMDR9 (9.7%), preceded by LGMDR1/2A (24.7%), LGMDR2/2B (23.8%), and sarcogycanopathies LGMDR3-5/2C-E (20%), among which the predominant one is LGMDR3/2D (46.7%) [21].

The most common variant in Europe associated with LGMDR9 is c.826C > A, located in exon 4 of the *FKRP* gene, which results in the replacement of a Leucine with an Isoleucine at position 276 (p.L276I) [22,23,24,25]. The high frequency of this variant, which falls within a common haplotype among populations of European origin, suggests a founder effect and a probable selective advantage [26].

LGMDR9 patients homozygous for p.L276I show a less severe phenotype and a later disease onset than patients in whom the same variant is present in compound heterozygosity [27,28]. The severity of the phenotype correlates with the variation (alteration) of the second allele [29].

However, intrafamilial and interfamilial phenotypic heterogeneity has been reported [30].

Patients with compound heterozygosity for a null allele and a silent variation have been described [30]. No patient homozygous for *FKRP* null alleles has been found, suggesting that the complete lack of FKRP might result in embryonic lethality [31].

Moreover, asymptomatic carriers of homozygous mutations in the FKRP gene and carriers manifesting LGMDR9 have been identified [22].

In the last decade, several FKRP mutant animal models have been developed showing the broad phenotypic spectrum observed in patients [32].

Mouse models carrying the common human variant p.L276I in homozygosis show a phenotype resembling the features of LGMDR9 as mild progressive myopathy, increased muscle regeneration, fibrosis, and a reduction in glycosylation of a-dystroglycan. Mice knockout for *FKRP* or homozygous for nonsense variants are embryonic lethal [33,34]. This is in agreement with what has been observed in humans.

Furthermore, zebrafish FKRP mutants that phenocopy WWS, MEB, or LGMDR9 diseases have been generated [35].

The great phenotypic heterogeneity observed in LGMDR9 patients could be due to different levels of glycosylation of α-DG, the more or less serious effects of the variants on protein activity, the loss of localization of FKRP in the Golgi apparatus, and its retention in the endoplasmic reticulum. The mutated protein may have a shorter half-life than the wild type as it is degraded through the proteasome complex [36].

To date, none of these hypotheses explains the reported phenotypic heterogeneity. It is likely that the observed clinical phenotypes are the result of many additional factors and molecular mechanisms [37].

Characteristic clinical signs at the onset of LGMDR9 are weakness in the proximal muscles of the lower limbs, waddling and tiptoeing gait, and frequent falls. Patients have difficulty running, getting up from the ground, and climbing stairs.

As the disease progresses, the muscles of the upper limbs are also involved, preventing the patient from lifting his arms or objects. Cretin kinase (CK) values in serum are strongly increased in the early stages of the disease and usually tend to decrease as the disease progresses, an expression of severe muscle atrophy and immobility. Other clinical signs are calf hypertrophy, macroglossia, joint contractures, pain, cramps, and muscle stiffness [29].

Cognitive functions, swallowing, and movement of the facial and neck muscles are generally not altered. Cardiac and respiratory problems are very frequent, especially if the disease is in an advanced stage [38,39,40].

Patients affected by LGMDR9 may present clinical signs that overlap with those of patients with the Duchenne/Becker phenotype. In particular, weakness of the proximal scapular and pelvic muscles, positive Gower’s sign, calf pseudohypertrophy, loss of ability to walk independently, elevated serum CK values, and cardiorespiratory involvement are common clinical signs [20,41,42].

For these reasons, we decided to conduct a molecular analysis of the *FKRP* gene in 153 patients from Southern Italy (Calabria) who had received a suspected diagnosis of Duchenne/Becker muscular dystrophy but who did not show pathological variants in the DMD gene.

## 2. Results

### 2.1. Sanger Sequencing

The molecular analysis of the entire 1.5-kilobase coding sequence of the *FKRP* gene was conducted on biological samples of 153 patients from Southern Italy (Calabria) by direct sequencing. All patients showed a Duchenne/Becker-like phenotype without confirmed genetic diagnosis.

Mutational screening of the FKRP gene allowed the identification of variants, previously reported as pathogenic [19,23,26,43,44] in 16 of 153 patients studied. The pathogenic variants identified with the highest frequency were p.L276I (c.826C > A) and p.R143S (c.427C > A). In particular, the p.R143S variant was identified in six patients (3.9%), while the p.L276I variant in four (2.6%) out of 153 patients. All patients with a genetic diagnosis were directed to specialized referral centers for accurate genetic counselling.

We report all the variants associated with LGMDR9 identified to date in Italian patients in Table 1.

Furthermore, mutational screening of the FKRP gene using Sanger sequencing conducted on patients from Southern Italy allowed the identification of four frequent polymorfic variants, already reported by Boito et al., 2005, Table 2. Of the four FKRP variants, three were silent nucleotide changes: c.135C > T (p.A45A), c.249C > T (p.A83A), c.192C > T (p.P64P), and one was located in the 5′untraslated region (−34C > T).

No correlation was observed between the type of variation identified and the clinical characteristics of the patients. The patients show a variable age of LGMDR9 onset (from 3 to 60 years of age) and a more or less severe progression of the disease. Serum Creatin kinase levels were very high. In some cases, cardiac and respiratory involvement is reported. All patients had normal cognitive function.

All information about them was derived from medical records.

We decided to focus our attention on the two variants p.R143S and p.L276I identified in our cohort with the greatest frequency. In particular, we decided to investigate the probable consequences of the p.R143S variant on protein function through in silico analyzes to support the probable pathogenic role of the variant, which is still up for debate. The p.R143S variant is frequently founded in single copies in patients affected by LGMDR9. This is in contrast to the autosomal recessive transmission of the LGMDR9.

### 2.2. Functional Prediction of the Missense Variant in the FKRP Gene (c.427C > A, p.R143S)

PolyPhen-2, Panther, and Mutation Taster in silico analyzes gave a score associated with a probable deleterious effect of the p.R143S (c.427C > A) amino acid substitution in exon 4 of the FKRP gene, Table 3.

### 2.3. Multi-Species Alignment

**Figure 1:**
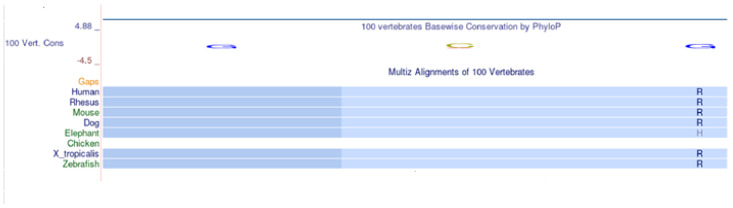
Multi-species alignment obtained by UCSC Browser. The symbol R (blue) indicates Arginine in position 143 conserved in Vertebrates.

The non-synonymous variant c.427C > A identified by Sanger sequencing determines the replacement of an Arginine in position 143 with a Serine (p.R143S). Arginine 143 is a conserved amino acid in evolution, as shown in the multispecies alignment, Figure 1.

### 2.4. FKRP 3D Structure Analysis

**Figure 2:**
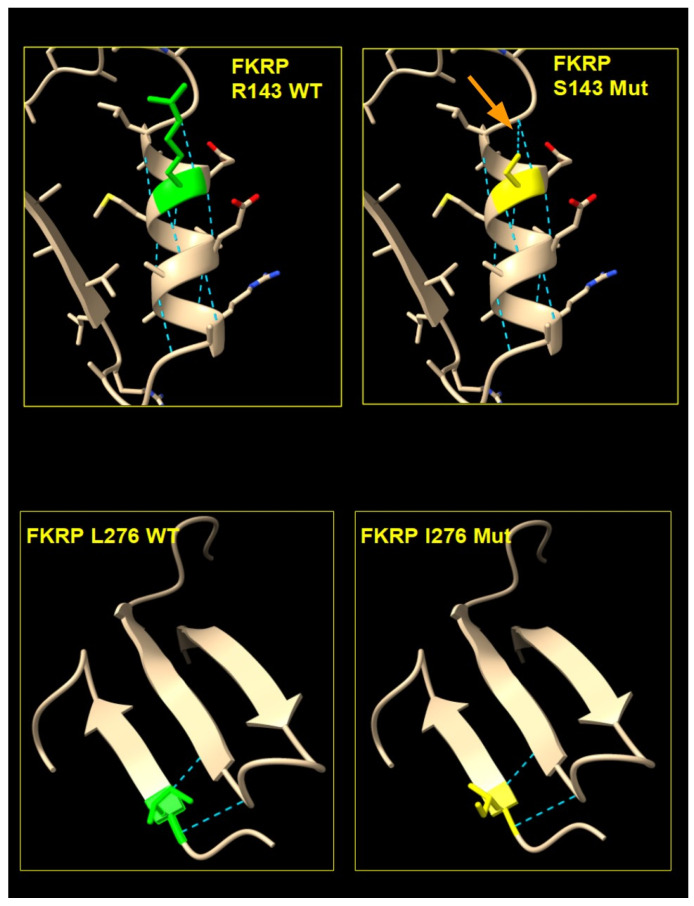
Three-dimensional structure model of mutated and wild-type FKRP. In green are indicated the amino acid residues of the wild type of Arginine (R) in position 143 and Leucine (L) in position 276 (**left** panels). In yellow are the variants Serine (S) in position 143 and Isoleucina (I) in position 276 (**right** panel). The light blue dashed lines indicate the hydrogen bonds, and the orange arrow indicates the new hydrogen bond in the mutated S143.

We obtained the structure of the FKRP 3D model by AlphaFold. The analyzed p.R143S and p.L276I variants are located in a region that has a very high confidence score (pLDDT > 90). In the wild-type proteins, the Arginine residue at position 143 belongs to the H3 alpha-helical motif, while the Leucine residue at position 276 participates in the formation of a beta sheet with adjacent beta strand motives, (Figure 2).

In the mutated proteins, the replacement of an Arginine with a Serine in position 143 of the FKRP protein determines the formation of a new hydrogen bond between the Serine 143 and the Glycine 139 residues, while no new bonds were observed in the case of the I276 mutated protein, (Figure 2).

**Figure 3:**
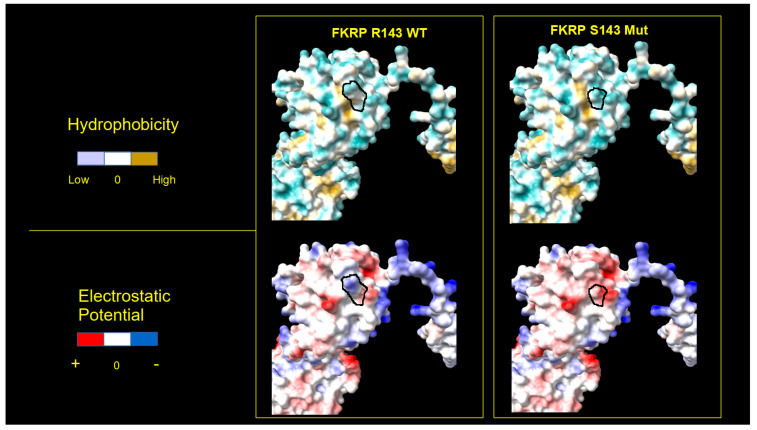
Three-dimensional surface of the R143S mutated and WT FKRP. Three-dimensional representation of electrostatic (**bottom**) and hydrophobic (**upper**) protein surfaces. Left panels show the chain with the Arginine (R) residue in position 143 (WT); right panels show the chain with the Serine (S) variant residue 143. The black closed line indicates the surface subtended by the 143 residues. Color scale is reported on the left side of panels.

**Figure 4:**
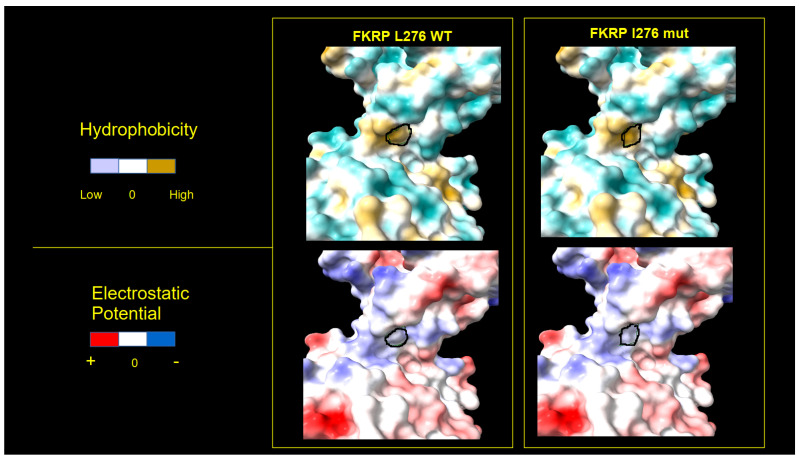
Three-dimensional surface of the L276I mutated and WT FKRP. Three-dimensional representation of electrostatic (**bottom**) and hydrophobic (**upper**) protein surfaces. Left panels show the chain with the Leucine (L) residue in position 276 (WT); right panels show the chain with the Isoleucine (I) variant residue in position 276. The black closed line indicates the surface subtended by the 276 residues. Color scale is reported on the left side of panels.

Furthermore, we analyzed the surface of the WT and mutated proteins using the electrostatic potential (Coulomb) and the hydrophobic features of the residues, detecting important differences between the mutated and WT 3D structures. The 3D structure of p.L276I, which derives from a pathogenic variant associated with LGMDR9, shows a lower degree of protein surface changes compared to p.R143S, Figure 3 and Figure 4.

### 2.5. MD Analysis

**Figure 5:**
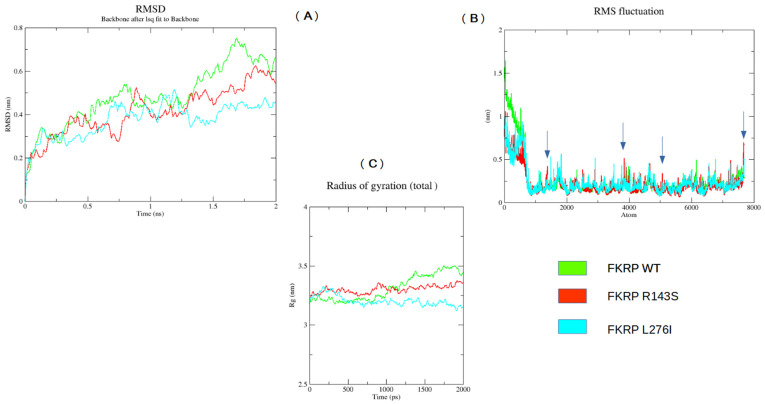
GROMACS Backbone Analysis. (**A**) Root-Mean-Square-Deviation RMSD; (**B**) Root-Mean-Square-Fluctuation RMSF; (**C**) Radius of gyration Rg. The values were calculated with respect to the Cα-atom. FKRP WT values are shown in green, while values for the p.R143S and p.L276I variants are shown in red and light blue, respectively. Arrows in (**B**) indicate some RMSF values of the p.R143S variant clearly different from WT.

We studied the effects of the amino acid variations on protein stability and function by Molecular Dynamic (MD) simulation. MD analysis was performed for a short time interval (2 ns). It was possible to observe appreciable differences in the trends of Root-Mean-Square Deviation (RMSD), Root-Mean-Square-Fluctuation (RMSF), and Radius of Gyration of the p.R143S and p.L276I variants compared to the respective wild-type chains, Figure 5.

In particular, starting from around 1.25 ns, the RMSD of the R143S and L276I variants showed deviations compared to the wild-type chains of 0.2 nm and 0.3 nm, respectively (Figure 5A). Appreciable was also the variation in the average fluctuation of the p.R143S variant for atom numbers 1380, 2517, 2902, and 3853 (Figure 5B).

Furthermore, the trend of the Radius of Rotation over time appears almost constant for the p.R143S variant, while that relating to the p.L276I shows a very varied trend over time. Both differ by wild type, Figure 5C.

### 2.6. Principal Component Analysis-PCA

We tested the stability of FKRP protein with the p.R143S and p.L276I variants compared to the wild-type protein by principal component analysis (PCA). PCA allows us to have information regarding the most influential internal movements of the protein during the simulation.

**Figure 6:**
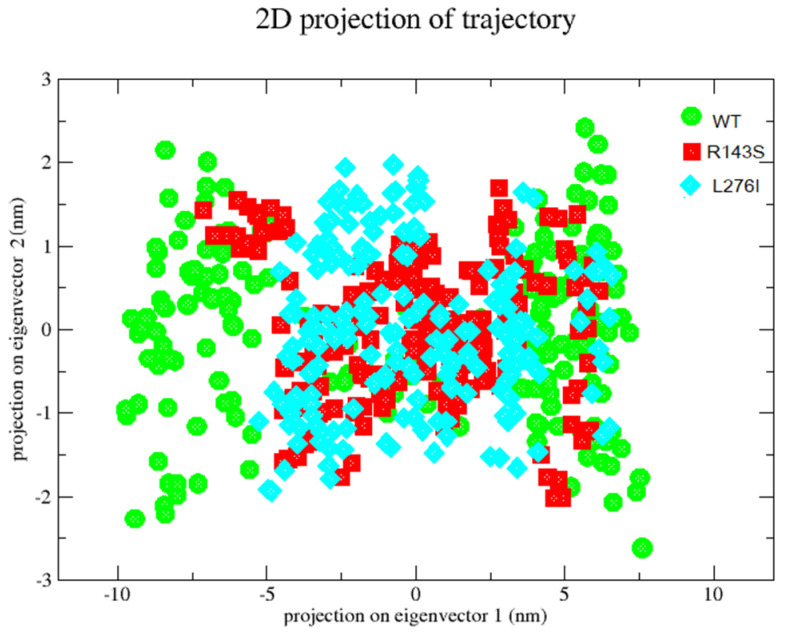
PCA of FKRP protein with variants and wild type. Projection of Cα-atoms along the principal two eigenvectors is shown. FKRP protein WT is shown in circle green; FKRP protein with p.R143S and p.L276I variants in square red and diamond light blue, respectively.

We used the first three eigenvectors. Both mutant proteins cover a smaller area than the wild type along the first two eigenvectors, Figure 6. The p.R143S variation showed less movement but slightly higher amplitude than the L276I. The latter showed a more restricted distribution of movements, especially in a range poorly covered by the wild-type protein. The data suggest a greater difficulty in movement of the two variants compared to the wild-type protein.

## 3. Discussion

Fukutin-Related Protein (FKRP) is a Golgi-resident glycosyltransferase involved in the post-translational modification process of dystroglycans. It transfers a ribitol-5-phosphate group (Rbo5P) to cytidine ribitol-5-phosphate (CDP-Rbo5P) to enable the formation of O-mannosylglycan, a key portion of α-dystroglycan (α-DG) [47] essential for glycosylation.

Defects in the O-mannosylglycan of α-DG have been associated with some forms of muscular dystrophies defined as α-dystroglycanopathies [48]. Muscular dystrophy patients may show on muscle biopsy a secondary reduction in some of the DGC proteins or of dystrophin itself. However, experimental data indicate that the reduction in glycosylation of α-DG does not correlate with the severity of the pathology [49]. This suggested the existence of other FKRP targets.

Molecular analysis conducted by direct sequencing of the entire coding region of the FKRP gene allowed us to identify causative variations of LGMDR9 in 16 patients (6 women and 10 men).

The p.L276I variant, reported with the greatest frequency in patients of European origin, was identified in four patients in homozygosity or compound heterozygosity with the p.P89L or p.A114G variant. This last variant in homozygosity was previously reported in a patient affected by Muscle–Eye–Brain disease [17] and compound heterozygosity in a Caucasian patient with Congenital Muscular Dystrophy and Walker–Warburg syndrome, respectively [14,44]. The p.P89L variant was previously reported in compound heterozygosity with the p.L276I variant in a French patient with a severe DMD-like phenotype [43].

The p.R143S variant (c.427C > A), first reported by Brockington et al., 2001 [14], was identified in 3.9% of our patients. We could not find the second mutated allele in any of the patients carrying p.R143S in agreement with what was reported in Italian, Spanish, and American patients [14,23,45,50,51,52].

In particular, of the approximately 18 patients previously reported, only three patients were homozygous or compound heterozygous for the p.R143S variant. Interestingly, all described patients were affected by muscular dystrophy with infantile and mild late-onset presentations (onset from 2 to 59 years of age); the serum CK levels were considerably elevated, and, in some cases, death occurred in the first months of life [23]. Therefore, the above-mentioned authors hypothesize that the second allele may reside in non-coding portions of the FKRP gene or that diallelic inheritance may exist [14,23,45]. Contrasting results were reported by Navarro-Cobos and Mamelona [20,53]. The identification of a single mutated allele is particularly frequent in patients with mutations in the CAPN3 (Calpain-3), DYSF (Dysferlin), and FKRP genes, as well as large intra- and inter-familial variability [45,54].

A probable pathogenic role of the p.R143S variant is suggested by PolyPhen-2 v2, Panther19.0, and MutationTaster2021 prediction software. Therefore, we have evaluated, in support of such a role in the onset of LGMDR9, the 3D model of the FKRP structure by AlphaFold2 and conducted a Molecular Dynamics (MD) study.

The 3D model of the FKRP structure, obtained by AlphaFold, shows that the replacement of the Arginine in position 143 with a Serine determines the formation of a new hydrogen bridge intra-helical, involving the Glycine residue at position 139 (Figure 2). Moreover, the amino acid residue change alters significantly the surface electrostatic potential and hydrophobicity in the mutated region with respect to the wild-type FKRP, Figure 3 and Figure 4.

Finally, we evaluated the involvement of the p.R143S and p.L276I variants on protein stability and function by the MD study, and we have found a pronounced alteration of molecular dynamics. In particular, the Root-Mean-Square Deviation (RMSD), Root-Mean-Square-Fluctuation (RMSF), and Radius of Giration (Rg) indicate appreciable differences of the p.R143S and p.L276I variants compared to WT, resulting in a probable loss of FKRP protein flexibility. The PCA plot also shows that the essential subspace occupied by the variants is narrower compared to the WT. This subspace is particularly important for its activity since the FKRP protein is organized in a homotetramer [5].

A crystallographic study suggested an essential role of the stem domain for FKRP enzymatic activity, participating in the O-Mannosyl glycan M3 recognition mechanism of the substrate [5]. Interestingly, p.R143S and p.L276I variants are located in the stem domain of the FKRP protein. The data obtained in silico from 3D structural and MD analyzes, allow us to assert that the p.R143S variant shows conformational and dynamic alterations similar to the well-known pathological p.L276I variant and that the p.R143S variant could therefore play a pathogenic role even if present in a single dose, influencing the normal glycosylation process.

Finally, we believe that the great inter- and intra-familial variability described in LGMDR9 could be due to other variants in different genes with additive and/or modifying effects on the phenotype, as reported in the literature [23].

Given the great variability and no clear correlation between phenotype and genotype, it is important to conduct a genetic screening population specific to identify affected patients early and provide adequate pharmacological treatments, appropriate family planning, counseling, and monitoring of the disease.

## 4. Materials and Methods

### 4.1. Patients

The study was conducted on 153 patients with a suspected diagnosis of Duchenne/Becker muscular dystrophy. Previous studies conducted on these samples had not highlighted alterations in the DMD gene responsible for Duchenne/Becker muscular dystrophy.

The patients included 41 women and 112 men (aged between 5 and 84 years) belonging to the same geographical area (Calabria). All subjects gave informed consent for the diagnostic investigation with explicit consent to future uses for research purposes, according to the Declaration of Helsinki.

### 4.2. DNA Extraction

Genomic DNA was extracted from peripheral venous blood using the “Salting Out” method [55].

A total of 40 mL Lysis Buffer (0.32 M Sucrose, 10 mM Tris-HCl pH 7.5, 5 mM MgCl2, and 1% Triton X-100) was added to 5 mL of blood. The sample was vortexed and centrifuged for 10′ at 3000 rpm at 4 °C. The supernatant was eliminated, and 5 mL of Fisio Buffer (NaCl 0.075 M, EDTA 0.025 M) was added to the pellet, vortexed, and centrifuged for 10′ at 3000 rpm at room temperature. The process was repeated a second time. At the end, the supernatant was eliminated and the pellet resuspended in 3 mL of Buffer A (Tris-HCl pH 8.0 10 mM, EDTA 2 mM), 100 μL of SDS 10% (AppliChem GmbH, Darmstadt, DE, Germany), and 30 μL of Proteinase K (Promega, Madison, WI, USA). The sample was vortexed and incubated in a thermostated bath at 65 °C for 1 h (or at 37 °C overnight). At the end of the pre-established time, the sample was vortexed with the addition of 500 μL of 6 M NaCl and centrifuged at 3000 rpm for 10′ at room temperature. The supernatant was aspirated, transferred to a 15 mL falcon, and Isopropanol was added up to a volume of 10 mL, shaken gently, and centrifuged at 3000 rpm for 10′ at room temperature. The supernatant was eliminated. The DNA pellet was washed with Ethanol at 70% (about 10 mL) and centrifuged for 10′ at 3000 rpm at room temperature. At the end of the centrifugation, the ethanol was completely eliminated, and the extracted DNA was resuspended in TE 1x pH8.0 (Tris-HCl pH 8.0, 0.01 M; EDTA pH 8.0, 0.001 M) and incubated in a thermostated bath at 65 °C for one hour.

Some DNA samples were extracted by Automatic Extraction using the EZ ADVANCED XL (Qiagen, Hilden, DE, Germany).

### 4.3. DNA Assay

DNA assay was performed using the Nano Drop 1000 (Thermo Fisher Scientific, Waltham, MA, USA). The amount of DNA sample used for the assay was 1 μL. We evaluated the 260 nm/280 nm absorbance ratio to test its purity.

### 4.4. Molecular Analysis of the FKRP Gene

The molecular-genetic investigation was conducted at the Institute for Biomedical Research and Innovation (IRIB), National Research Council CNR, Mangone (CS), Italy.

The extracted DNA was amplified by Polymerase Chain Reaction (PCR) using ProFlex (Thermo Fisher Scientific, Waltham, MA, USA).

The entire coding region of 1.5 kb of the FKRP gene was split into multiple fragments by sets of partially overlapping primers. The primers used to amplify these exonic fragments and part of the intronic regions are indicated in Table 4.

### 4.5. PCR Reaction

Exonic fragments were amplified using ProFlex (Thermo Fisher Scientific, Waltham, MA, USA). We used 50 ng/μL genomic DNA, 200 μM dNTPs, 10 pmol/μL of each primer, and 0.25 μL (5 U/μL) TaqAdvantage GC Genomic LA (Takara Bio USA, Inc., San Jose, CA, USA) in a final volume of 25 μL. The thermal profile used was the following: Predenaturation 94 °C for 1′; Denaturation 94 °C for 30′; Annealing 60 °C * for 30′; Extension 72 °C for 1′ for 35 cycles. Final delay 72 °C for 5′.

Each amplified exonic fragment was purified using CleanSweep PCR Purification Reagent (Thermo Fisher Scientific, Waltham, MA, USA). A total of 2 μL of enzyme was added to 5 μL of the PCR sample and incubated for 15′ at 37 °C or 15′ at 80 °C.

The TM value reported above was used to amplify the 5F-5R fragment; the 1F-2R fragment was amplified with a TM of 56 °C; finally, the 3F-4R fragment with a TM of 53 °C.

### 4.6. Sequence Reaction

Each amplified and purified exonic fragment was sequenced using the ABI PRISM 3130 XL Genetic Analyzer (Thermo Fisher Scientific, Waltham, MA, USA). A total of 1 μL of Big Dye Terminator Ready Reaction Mix v.3.1 was added to 7 μL of sample previously purified with enzyme 1.1 (Thermo Fisher Scientific, Waltham, MA, USA), 3.2 pmol/μL primer (Forward or Reverse) in a final volume of 10 μL.

We used the following thermal profile for the sequence reaction: Predenaturation 96 °C for 1′; Denaturation 96 °C for 10′; Annealing 50 °C for 5′; Extension 60 °C for 4′; 25 cycles.

The samples were purified using Centri-Sep 96 (Applied Biosystems) for Princeton Separations. A total of 3.5 μL of each sample with 12 μL of formamide was loaded onto a 96-well plate (MicroAmp Optical 96-Well Reaction Plate, Thermo Fisher Scientific, Waltham, MA, USA) and processed on an ABI PRISM 3130 XL Genetic Analyzer capillary sequencer (Thermo Fisher Scientific, Waltham, MA, USA).

### 4.7. FKRP 3D Structure

The 3D structure of FKRP was analyzed using AlphaFold2 3D model data. This Artificial Intelligence system, developed by DeepMind, is capable of accurately predicting a protein’s 3D structure from its amino acid sequence [56,57]. We used the ChimeraX software version 1.1 “https://www.cgl.ucsf.edu/chimerax/ (accessed on 24 November 2022)” to view and analyze the 3D structure codified into the PDB file AF-Q9H9S5-F1.pdb for both wild-type and mutated FKRP proteins. The AlphaFold2 database is available online at “https://alphafold.ebi.ac.uk/ (accessed on 24 November 2022)”, selectable also from the Uniprot database at https://www.uniprot.org/ (accessed on 24 November 2022).

### 4.8. Molecular Dinamic Simulation (MD)

MD simulations were performed using GROMACS 2023 homebrew on WT and mutated proteins. The GROMACS input FKRP file was the AF-Q9H9S5-F1.pdb obtained from the AlphaFold protein database (AlphaFold DB) and created by an AI model developed by AphaFold [56,57]. We decided to use this model since it includes all the amino acids of the protein. In the experimental one, however, the transmembrane and part of the luminal portion are missing. The same file was used to introduce the mutations p.R143S and p.L276I using the ChimeraX software (https://www.cgl.ucsf.edu/chimerax) (accessed on 16 March 2023). The simulation was performed on the entire protein structure for both WT and mutated proteins in order to obtain overall indications of molecular dynamics for each of them. Subsequently, comparing the data, we estimated the deviations in the dynamics of the mutated proteins when compared to the WT one. The force field selected for the simulation was the OPLS-AA/L. MD preparation was performed by adding TIP3P water models inside a cubic box. Subsequently, Na^+^ and Cl^−^ ions were added to neutralize the system, and 5000 steps using the steepest descent method were performed to minimize them. The system was equilibrated by the constant Number of particles, the Volume and Temperature ensemble (NVT), and then, by the constant Number of particles, the Pressure and Temperature ensemble (NPT). Finally, the MD simulation was set to run for 2 ns. The Root Mean Square Deviation (RMSD), the Root Mean Square Fluctuation (RMSF), the Radius of gyration and the principal component analysis (PCA) were obtained by GROMACS tools. MD data analysis was performed by using GRACE, a graph plotting tool software “https://plasma-gate.weizmann.ac.il/Grace/” (accessed on 16 March 2023).

## 5. Conclusions

Patients affected by a severe/mild course of LGMDR9 may present a Duchenne/Becker-like phenotype, sometimes with a secondary dystrophin reduction on muscle biopsy.

Our study reveals that patients from South Italy with a Duchenne/Becker-like phenotype, without dystrophin gene rearrangement, often show mutations in the gene encoding FKRP (10.4%). The p.R143S is a recurrent variant in patients studied, probably due to a founder effect. We failed to find the second mutated allele in patients carrying p.R143S in agreement with what previously reported.

PolyPhen-2, Panther, and Mutation Taster prediction software suggests a likely pathogenic role of the p.R143S variant.

We evaluated, in support of its probable role in the onset of LGMDR9, the 3D structure of the FKRP AlphaFold model and conducted a Molecular Dynamics study. The data obtained showed that the conformational change of FKRP protein, induced by the replacement of an Arginine with a Serine in position 143, determines alterations of the surface electrostatic potential and hydrophobicity.

Furthermore, data obtained by MD suggest that the p.R143S variant results in reduced chain flexibility when compared to the WT, and in addition, data from principal component analysis highlight a reduction in the subspace occupied that is particularly important for its o-Mannosyl glycan M3 substrate recognition activity. The p.R143S variant shows a molecular dynamic similar to the well-known pathological p.L276I variant reported with the greatest frequency in patients of European origin.

The results obtained suggest that the p.R143S variant could play a pathogenic role even if present in a single dose.

However, we believe that the large inter- and intra-familial variability described in LGMDR9 could be due to other variants in different genes with additive and/or modifying genes on the phenotype.

Our study highlights the importance of considering LGMDR9 in the differential diagnosis of dystrophinopathies in Calabria for the rapid identification of patients, an essential element for the development of new and effective treatments.

## Figures and Tables

**Table 1:** Causative variants of LGMDR9 identified so far in Italian patients. Colored in blue are the p.L276I and p.R143S variants most represented in our sample and in common with previously published studies.

Boito et al., 2005 [23]	Magri et al., 2005 [21]	Guglieri et al., 2008 [45]	Mercuri et al., 2009 [46]	Our Work
L276I	L276I	L276I	W231C	L276I
R143S	L172I	R143S	P315S	R143S
P462S	L319R	L319R		P89L
P358L		V160		A114G
R244H		P104S		S115L

**Table 2:** Polymorfic variants identified in patients from Southern Italy (Calabria).

Variants	dbSNP
−34C > T	rs3201779
A83A	rs149030303
A45A	rs2287717
P64P	rs111754012

**Table 3:** Functional prediction of the missense variant in the FKRP gene (c.427C > A, p.R143S).

Tool	Effect	Score/Reliability Index	Link
PolyPhen2	Deleterious	0.93	http://genetics.bwh.harvard.edu/pph2/ (accessed on 28 September 2022)
Panther	Deleterious	0.5	http://www.pantherdb.org/tools/ (accessed on 28 September 2022)
Mutation Taster	Deleterious	0.99	https://www.mutationtaster.org/ (accessed on 28 September 2022)

**Table 4:** Forward and Reverse primer sequences.

EXON	PRIMER
FKRP-EX4-1F	5′ AAAGGGAATTGAGAAAGAGC 3′
FKRP-EX4-2R	5′ CCGAGAGGTTGAAGAGGT 3′
FKRP-EX4-3F	5′ AGTTTGTGGCCCTAGTACCT 3′
FKRP-EX4-4R	5′ CCTTCTCCCATACGAAGC 3′
FKRP-EX4-5F	5′ TGGAGGCTGCGGGCGTGCGCTACTG 3′
FKRP-EX4-5R	5′ GCTCACACAGAGCTTCTCC 3′

## Data Availability

All datasets generated or analyzed during the study are available upon request.
